# Fibrous Dysplasia of the Palate: Report of a Case and Review of Palatal Swellings

**DOI:** 10.1155/2012/179853

**Published:** 2012-09-17

**Authors:** Balasundari Shreedhar, Mala Kamboj, Nishant Kumar, Sameera Shamim Khan

**Affiliations:** Department of Oral Pathology and Microbiology, Career Post Graduate Institute of Dental Sciences & Hospital, Ghailla, Lucknow 226003, India

## Abstract

Fibrous dysplasia is a benign fibroosseous lesion characterised by the replacement of normal bone by excessive proliferation of cellular fibrous connective tissue which is slowly replaced by bone, osteoid, or cementum-like material. It causes bone pain, deformities, and pathological fractures. Fibrous dysplasia (FD) is a sporadic benign skeletal disorder that can affect one bone (monostotic form) or multiple bones (polyostotic form). In this paper, we present case of a monostotic fibrous dysplasia on the hard palate of 14-year-old girl and a tabular review of common palatal swellings.

## 1. Introduction

Fibrous dysplasia (FD) is a disturbance of bone metabolism that is classified as a benign fibroosseous lesion in which the fibrous connective tissue containing abnormal bone replaces normal bone [1]. Fibrous dysplasia is a sporadic benign skeletal disorder that can affect one bone (monostotic form) or multiple bones (polyostotic form). Gender prevalence of FD is equal. The monostotic form is more common and affects 20–30 years of age while the polyostotic form has its onset mainly in children younger than 10 years of age [2].

FD is attributed to GNAS1 (guanine nucleotide-binding protein, alpha-stimulating activity peptide 1), gene mutation resulting in the abnormal proliferation and differentiation of preosteoblast [3]. Malignant changes are relatively low [3, 4]. A systemic disease named “McCune-Albright syndrome” is characterised by polyostotic fibrous dysplasia, cafe-au-lait skin hyperpigmentation, and endocrinopathy [3]. Another type identified by Daves is craniofacial fibrous dysplasia which involves two or more facial and cranial bones. The distribution of fibrous dysplasia is as follows 74% monostotic, 13% polyostotic, and 13% craniofacial form.

We hereby report case of a 14-year-old girl with fibrous dysplasia on the palate and at the same time we present a review of common palatal swellings in tabular form (Table 1) which could be a cause of confusion at the time of diagnosis.

## 2. Case Report

**Figure 1:**
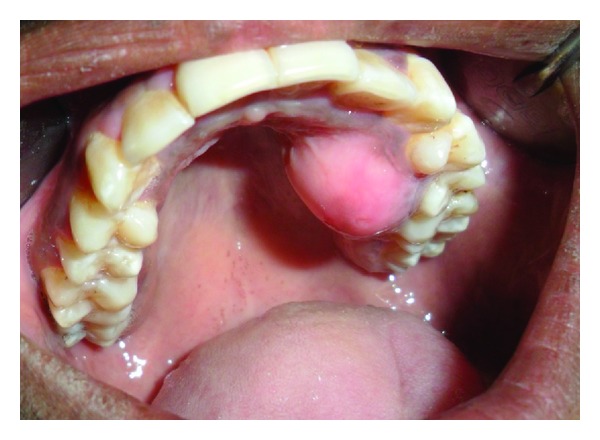
A clinical view of diffuse, bony hard swelling in the maxillary left region of hard palate.

**Figure 2:**
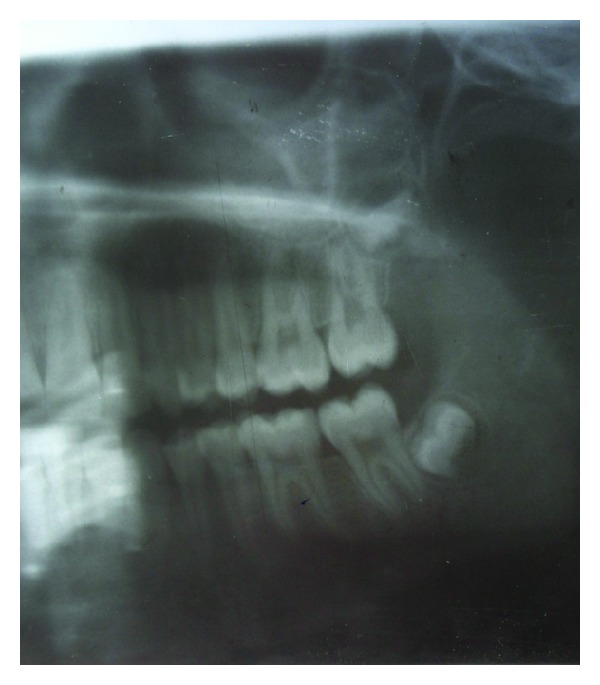
An orthopantomogram showing mottled unilocular radiolucency.

A 14-year-old girl visited the college with the chief complaint of swelling in the upper left back region of jaw. The patient was healthy and gave no history of systemic diseases or drug allergies. On clinical examination a diffuse, bony hard nontender swelling in the maxillary left region of hard palate was seen measuring approximately 2.5 × 3.3 cm and extending from left 1st premolar to 2nd molar region (Figure 1). On orthopantomogram (OPG), a mottled unilocular radiolucency was visible in the affected area (Figure 2).

**Figure 3:**
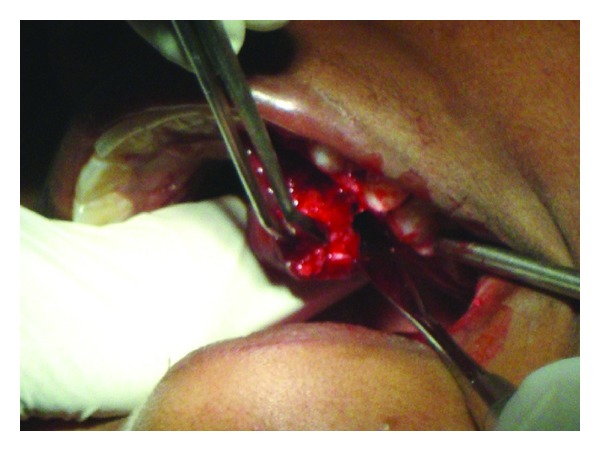
An excisional biopsy of the lesion.

**Figure 4:**
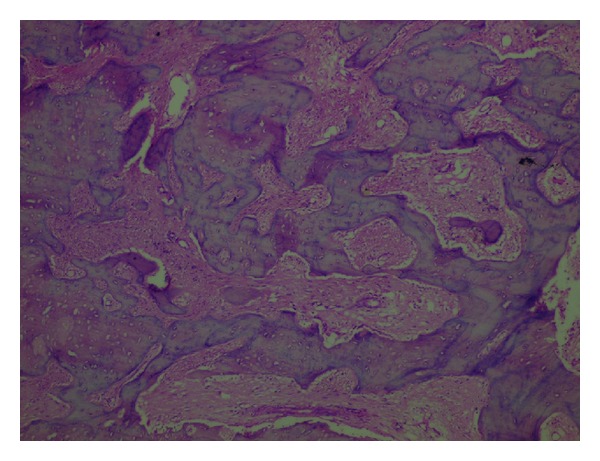
A photomicrograph showing “C” or “Chinese letter” shaped bony trabeculae in a highly cellular connective tissue (H/E, 10X).

**Figure 5:**
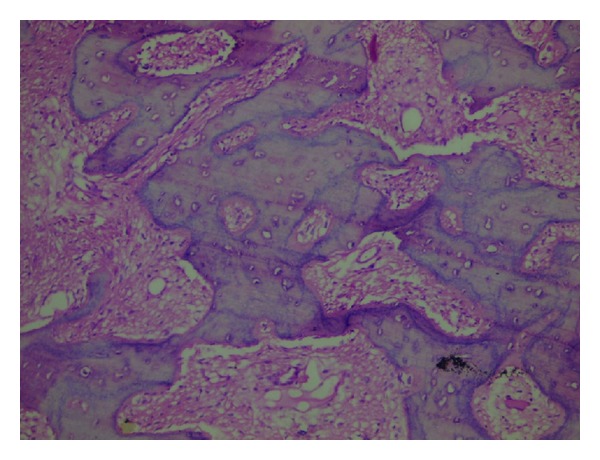
A photomicrograph showing trabeculae with presence of osteocytes and absence of peripheral rimming of osteoblasts (H/E, 20X).

The lesion was surgically excised (Figure 3) and the tissue was sent for histopathological examination. The sections revealed “C” shaped/Chinese letter-shaped bony trabeculae in a highly cellular connective tissue. The trabeculae showed presence of osteocytes and absence of peripheral rimming of osteoblasts suggestive of fibrous dysplasia (Figures 4 and 5). 

The patient is under followup for the last 6 months and seems to be disease free.

## 3. Discussion

Fibrous dysplasia is defined as “a benign lesion, presumably developmental in nature, characterised by the presence of fibrous connective tissue with a characteristic whorled pattern and containing trabeculae of immature nonlamellar bone” [5]. Eversole defines fibrous dysplasia of craniofacial bones as “a benign, nonneoplastic intramedullary cellular proliferation of fibroblasts, with formation of irregular trabeculae of bone or ovoid calcifications that shows indistinct, nonencapsulated borders” [5].

Fibrous dysplasia is a hamartomatous condition or disorder of bone metabolism [3]. The monostotic form of fibrous dysplasia generally occurs in second decade of life. The craniofacial form occurs earlier and more severely, typically around 10 yrs of age, and then progresses throughout adolescence [6].

The other subtypes of polyostotic dysplasia show cafe-au-lait pigmented skin lesions and endocrinopathies when they are called Jaffe-Lichtenstein syndrome and McCune-Albright syndrome. The diagnosis of monostotic fibrous dysplasia could be based on clinical, radiographic, and histopathological findings [6]. Patient is usually seen with abnormal enlargement of jaw without pain or infection, “ground-glass” pattern seen in radiographic film, can be shifted close to film, and “Chinese character” or “fish bone” woven bone patterns seen in a microscopic view are also unique characteristics [3, 7].

Mirra supported a concept, which stated, “when a single bone (monostotic) is affected, it probably represents a forme fruste of the more severe form (polyostotic).” He stated that the craniofacial bones affected by fibrous dysplasia are in the following order that is frontal > sphenoid > ethmoid > maxilla > mandible > zygoma > parietal > occipital > temporal [5].

Treatment of bony lesions of fibrous dysplasia includes surgical and nonsurgical therapies. Surgical treatment in young-aged minor cases and biopsy with minor bony osteoplasty at affected site are adequate. In more severe cases complete excision with graft reconstruction may be possible [8].

 Useful biomarkers such as serum alkaline phosphatase and urinary hydroxyproline can be used to monitor response in the nonsurgical treatment of the disease rather for diagnosis [9, 10]. Therefore in this case the clinical features, radiographic features, and histopathological features were correlated to come to a final decision of fibrous dysplasia.

A tabular form of common palatal swellings (Table 1) has been added to rule out any confusion at the time of clinical diagnosis.

## 4. Conclusion

The fibrous dysplasia is significant for the dentists because it may affect the facial, cranial, and jaw ones leading to many deformities and dysfunctions. The cells of fibrous dysplasia are committed osteogenic cells with impaired capacity to form normal bone. The mutated protein not only affects osteoblasts but can also affect various hormone receptors leading to endocrinopathies and cafe-au-lait spots. Our case did not show any skin pigmentations or endocrinopathies. Malignant transformation occurs infrequently with reported frequencies ranging from 0.4–4%. Our case has not shown any malignant changes till date and the followup is still ongoing. In young-aged monostotic fibrous dysplasia cases, regular followup or minor bony osteoplasty at affected site is adequate for esthetic and functional purpose.

## Figures and Tables

**Table 1:** Differential diagnosis of common palatal swellings.

Serial number	Palatal swellings	Clinical features	Histopathological features
(1)	Mucus extravasation phenomenon	(i) Bluish nodule covered by epithelium (ii) Painful, seen in lower lip, buccal mucosa (iii) Adolescent and children common	(i) False cyst, circumscribed cavity in connective tissue and submucosa (ii) Wall is made up of lining of compressed fibrous connective tissue and fibroblast
(2)	Pleomorphic adenoma	(i) Solitary, firm asymptomatic mass covered by epithelium (ii) Malignant tumors may cause pain, paresthesia, or ulceration (iii) Young adults (iv) Most common intraorally in palate followed by tongue, upper lip	(i) Epithelial component forms duct and small cyst containing eosinophilic coagulum (ii) Small cellular nest, sheath of cells, foci of keratinising squamous or spindle cells, myoepithelial cells appear angular or spindle, rounded with eccentric nuclei and hyalinised eosinophilic cytoplasm
(3)	Non-Hodgkins Lymphoma	(i) Common in male older than 50 yrs (ii) Endemic geographical factor appears to influence development of non-Hodgkins lymphoma. (iii) Systemic symptoms fever, night sweats, weight loss, and fatigue (iv) Organ specific symptoms—shortness of breath, chest pain, cough, abdominal pain, bone pain	(i) Nodular or diffuse nodular pattern—neoplastic cells aggregate and large clusters of cells are seen (ii) Diffuse pattern is characterised by monotonous distribution of cells
(4)	Torus	(i) Asymptomatic bony hard swelling of hard palate, bony exophytic bone along lingual aspect of mandibular torpid (ii) Young adults	(i) Compact/cancellous bone
(5)	Neoplasm of maxilla or maxillary sinus	(i) Palatal swelling with or without ulceration, pain, or paresthesia (ii) Loosening of teeth or malocclusion (iii) Any age, rare	
(6)	Palatal abscess from periapical region	(i) Painful, pus-filled, fluctuant tumescence of hard palate, (ii) Reddish color associated with nonvital tooth	
(7)	Adenoid cystic carcinoma	(i) 5th and 6th decade of life, common in females (ii) Salivary glands most commonly involved are parotid, submaxillary, and accessory glands in palate and tongue (iii) Symptoms pain, facial nerve paralysis, and ulceration	(i) Composed of myoepithelial cells and ductal cells (ii) Morphologically 3 growth patterns cribriform—Swiss cheese or honey comb pattern (iii) Tubular pattern—lined by stratified cuboidal epithelium (iv) Solid pattern—group of cuboidal cells with tendency of duct or cyst formation
(8)	Abrikossoff's tumor	(i) Benign lesion affecting the mucous membrane of the upper aerodigestive tract (ii) Common site anterior part of the tongue (iii) Predilection for females, age range varies from 4 months to 89 years	(i) Necrosis, cells in fusiform strings, nuclear pleomorphism, large nucleus with vesicular core (ii) Increased mitotic activity (iii) Increased nucleus radius in relation to cytoplasm
